# Serum cystatin C levels are independently correlated with cognitive impairment in individuals with cerebral small vessel disease

**DOI:** 10.3389/fnins.2026.1781698

**Published:** 2026-04-28

**Authors:** Danheng Mo, Mingchun Li, Xihua Guo, Lu Zhou, Jianwen Yang, Tao Lei, Wenping Zou, Qiang Lei, Qing Wang, Chunguang Li, Xi Tao

**Affiliations:** 1 Department of Neurology, Zhujiang Hospital, Southern Medical University, Guangzhou, China; 2 Department of Neurology, Hunan Provincial People’s Hospital, Hunan Normal University, Changsha, Hunan, China; 3 Department of Neurology, Second Xiangya Hosiptal, Central South University, Changsha, Hunan, China; 4 Department of Neurological Rehabilitation, Hunan Provincial People’s Hospital, Hunan Normal University, Changsha, Hunan, China; 5 Vascular Depression Diagnosis, Treatment, and Rehabilitation Technology Innovation Center of Changsha, Changsha, Hunan, China

**Keywords:** biomarkers, cerebral small vessel disease, correlation, cystatin C, vascular cognitive impairment

## Abstract

**Background and purpose:**

Previous studies have shown that serum cystatin C (CysC) is associated with cerebral small vessel disease (CSVD) and that elevated CysC levels are linked to an increased risk of cognitive impairment in the elderly. However, whether CysC is specifically associated with cognitive impairment in patients with CSVD remains unclear.

**Method:**

A total of 334 CSVD patients with available demographic, blood biomarker, and brain imaging data were included. Patients were divided into vascular cognitive impairment and normal cognition groups. Univariate analysis was used to compare baseline data, blood biomarkers, imaging features, and behavioral scores between the two groups. Binary logistic regression was used to evaluate the diagnostic value of cystatin C for CSVD-related cognitive impairment.

**Results:**

Compared with the normal cognition group, the VCI group exhibited significantly elevated serum levels of CysC, homocysteine, urea nitrogen, creatinine, uric acid, fibrinogen, and D-dimer, along with a lower red blood cell count. The VCI group also showed a higher prevalence of severe periventricular white matter hyperintensity, severe deep white matter hyperintensity, severe total white matter hyperintensity, and brain atrophy. The combination of these eight blood biomarkers markedly improved the diagnostic performance for VCI (AUC = 0.672, 95% CI: 0.615–0.730, *p* < 0.001). Multivariate analysis revealed that elevated CysC levels (OR = 2.677, *p* = 0.041), age (OR = 1.067, *p* < 0.001), and severe total WMH (OR = 2.713, *p* < 0.001) were associated with CSVD-related cognitive impairment. After adjusting for confounding variables, serum CysC levels remained independently correlated with cognitive impairment (OR = 3.257, 95% CI: 1.192–8.899, *p* = 0.021).

**Conclusion:**

Serum CysC levels are independently associated with cognitive impairment in CSVD patients.

## 1 Introduction

Cerebral small vessel disease (CSVD) is defined as damage to the small vessels of the brain and encompasses a collection of related clinical, imaging, and pathological syndromes (Pantoni, 2010). The clinical manifestations of CSVD are diverse and mainly include asymptomatic CVSD, lacunar stroke and cognitive dysfunction. In recent years, with the in-depth exploration of the pathological mechanisms of small blood vessels, researchers have gradually noted the importance of CSVD in diseases such as cognitive impairment, acute cerebrovascular disease and dyskinesia (Wardlaw et al., 2019; Debette et al., 2019; Chen et al., 2021). Research on the early diagnosis of CSVD-related cognitive impairment (CSVD-CI) has focused mainly on the exploration of blood biomarkers, cerebrospinal fluid biomarkers, imaging biomarkers and artificial intelligence-assisted diagnosis techniques (Wang et al., 2025a; Wang et al., 2025b). The wide application of multimodal neuroimaging techniques, combined with the auxiliary analysis of artificial intelligence algorithms, has significantly increased the ability to identify CSVD features such as enlarged perivascular spaces, white matter hyperintensity (WMH) and cerebral microbleeds, providing an important basis for early intervention (Chen et al., 2024; Shahid et al., 2024; Jiang et al., 2022). However, this field faces significant limitations: neuroimaging is costly and relies on specialized equipment, thereby limiting its widespread adoption in primary care settings; furthermore, the sensitivity and specificity of many techniques remain suboptimal. In contrast, the detection of blood biomarkers is noninvasive, inexpensive, and repeatable and is thus easier to promote for clinical application.

Serum cystatin C (CysC), which is a member of the cysteine protease inhibitor superfamily, is the main endogenous cysteine protease inhibitor. It has diverse biological functions, contributes to normal physiological processes, including cell proliferation and astrocyte differentiation, and participates in pathological events such as neurodegeneration and inflammatory responses. Recent studies have revealed that CysC has multiple potential neuroprotective mechanisms, including inhibiting cathepsin B (CatB) activity (Kaur et al., 2010), blocking the mitochondrial apoptosis pathway by activating the PI3K/AKT pathway, enhancing neuronal antistress ability (Kaur et al., 2018), and directly binding to amyloid β-protein (Aβ) to regulate its metabolism and inhibit its oligomerization (Tizon et al., 2010). However, the involvement of CysC in the pathogenesis of Alzheimer’s disease is controversial (Ciccone et al., 2020). On the one hand, CysC plays a neuroprotective role by inhibiting Aβ aggregation through its binding to Aβ (Tizon et al., 2010); on the other hand, as a substrate of CatB, CysC competitively inhibits the CatB-mediated Aβ degradation pathway by inhibiting CatB activity (Sun et al., 2008). This contradiction has also been observed in clinical studies of cognitive impairment. Serum CysC levels are significantly elevated in patients with Alzheimer’s disease and vascular dementia (Wang et al., 2017), and these higher levels are also linked to a higher risk of mild cognitive impairment (Li et al., 2023). Studies of community-dwelling older adults have demonstrated that elevated CysC levels are correlated with not only concurrent cognitive impairment (Cui et al., 2020) but also a twofold greater likelihood of the development of cognitive decline over 7 years (Yaffe et al., 2008). Notably, even if a patient does not have clinical symptoms, elevated serum CysC levels are still significantly correlated with CSVD imaging markers (WMH, cerebral microbleeds, etc.) and total imaging load (Yao et al., 2022), suggesting that elevated CysC levels may be an early response to microvascular injury. In the dominant stage of CSVD, this correlation is further strengthened (Guoxiang et al., 2018; Oh et al., 2014), suggesting that elevated CysC levels in a pathological state may be a marker of compensatory mechanism failure or disease progression.

While prior studies have established associations among serum CysC levels, cognitive impairment, and CSVD imaging markers, a systematic investigation into the relationship of CysC levels with cognitive decline specifically within a diagnosed CSVD population is lacking. Consequently, this observational study aimed to elucidate the relationship between serum CysC levels and cognitive dysfunction in patients with CSVD and to evaluate its utility as a diagnostic biomarker for CSVD-CI.

## 2 Methods

### 2.1 Participant groups

**Figure 1:**
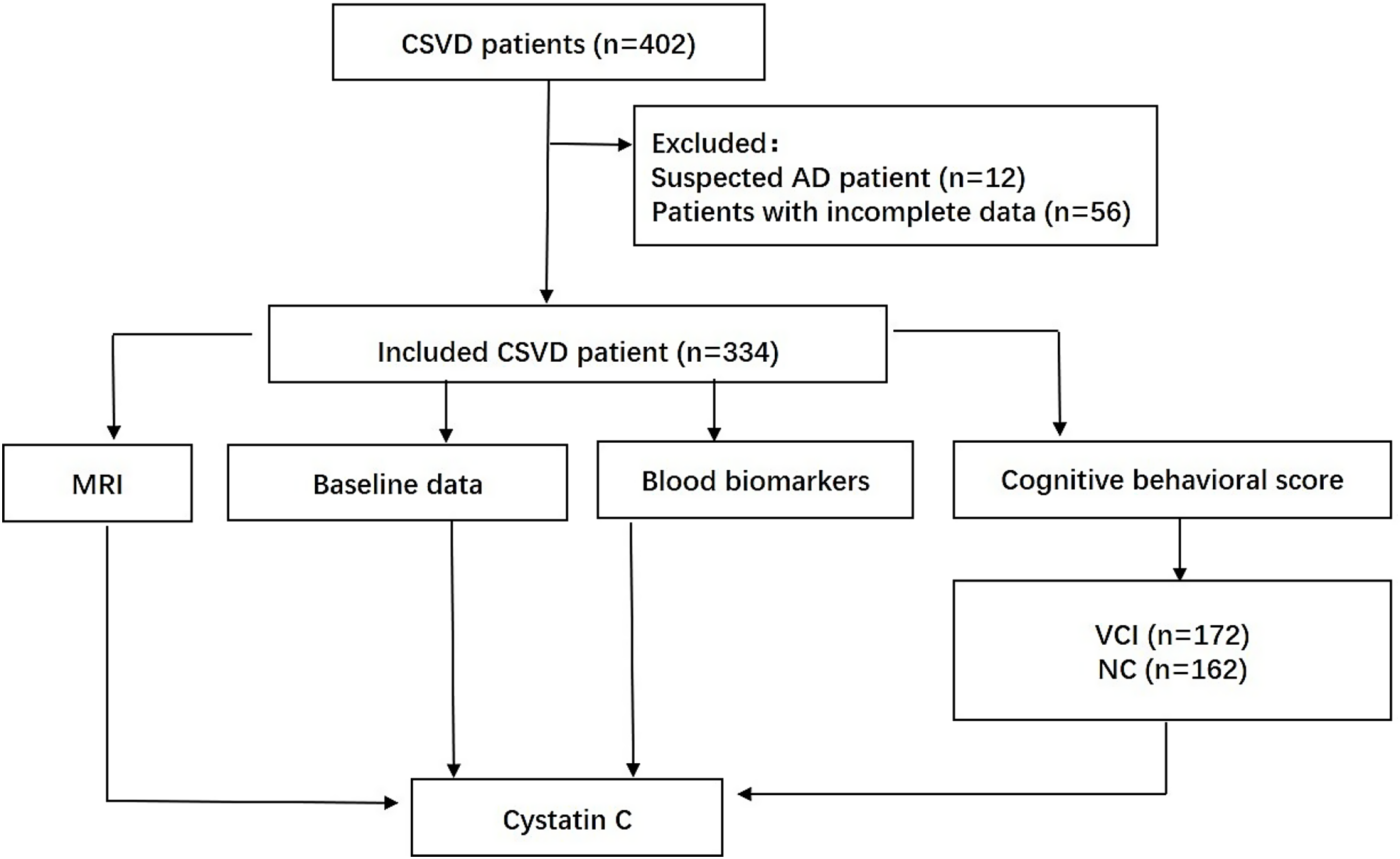
Study flow chart. CSVD, cerebral small vessel disease.

We conducted a retrospective, observational study. Data were collected from 402 CSVD patients at a single center between May 2020 and July 2021. After applying the exclusion criteria, 68 patients were removed for the following reasons: 56 for incomplete data records and 12 because of comorbid psychiatric diagnoses that compromised their capacity to complete the required evaluations. The resulting analytical sample thus consisted of 334 patients (Figure 1). These patients presented with various clinical symptoms, including but not limited to dizziness, headache, cognitive decline, anxiety, depression, and acute vascular events. All the patients underwent 1.5T brain magnetic resonance imaging (MRI), which confirmed the presence of at least one of the following imaging features: lacunar infarction, white matter hyperintensities, enlarged perivascular spaces, cerebral microbleeds, or brain atrophy. The final diagnosis of CSVD was established by experienced neurologists in accordance with established diagnostic criteria (Hu et al., 2021).

To be included, patients were required to satisfy the following criteria: (1) met the diagnostic criteria for age and vascular risk factors related CSVD in the “Chinese Consensus on Diagnosis and Therapy of Cerebral Small Vessel Disease 2021 (Hu et al., 2021),” (2) demonstrated clinical stability, as evidenced by stable vital signs, (3) had clear consciousness, and (4) provided sufficient cooperation for valid behavioral examinations. Patients were excluded from the analysis for any of the following reasons: (1) incomplete data, (2) a clear clinical diagnosis of nonvascular cognitive impairment (e.g., psychiatric disorders, Alzheimer’s disease, or other neurodegenerative diseases), (3) alanine aminotransferase levels > 200 U/L or estimated glomerular filtration rate (eGFR) < 30 mL/min/1.73 m^2^, (4) active systemic infection within 14 days prior to evaluation, or (5) severe aphasia that would invalidate cognitive testing. This research received approval from the Ethics Committee of the People’s Hospital of Hunan Province, Hunan Normal University (Approval No. [2025]-38). All participants provided written informed consent upon admission, agreeing that their anonymized medical data collected during hospitalization could be used for scientific research.

### 2.2 Clinical characterization

Baseline demographic and clinical data were obtained for all participants. The collected variables included age, gender, body mass index (BMI), years of education, and history of smoking and alcohol intake. We also determined the prevalence of key comorbidities, including stroke, hypertension, diabetes mellitus, and coronary heart disease. History of stroke was defined as a prior hospitalization for a cerebrovascular event. In contrast, histories of hypertension, diabetes mellitus, and coronary heart disease were based on a confirmed diagnosis documented within the medical record, regardless of whether it was established before or during the index admission. Years of education were recorded as the total years of formal education completed.

### 2.3 Cognitive behavioral assessment

The cognition and cognitive subdomains of all patients were assessed. Cognitive–behavioral assessments were performed by experienced senior physicians. The methodology for these assessments was based on the consensus research guidelines for the classification of vascular cognitive impairment (VCI) (Skrobot et al., 2016) and the Chinese guidelines for the diagnosis and treatment of VCI (Chinese Stroke Association Vascular Cognitive Impairment Subcommittee, 2024). We administered the simplified Chinese version of the Mini-Mental State Examination (MMSE) to conduct a global cognitive screening of the participants (Mellor et al., 2016). This examination was supplemented with the Montreal Cognitive Assessment (MoCA) for a more detailed evaluation (Mellor et al., 2016; Wang et al., 2019). The MMSE scale is based on the following cognitive impairment score thresholds according to educational background: illiterate, ≤ 17 points; 1–6 years, ≤ 19 points; and ≥ 7 years, ≤ 24 points (Li et al., 2016). A MoCA score of less than 26 points is considered to indicate cognitive impairment (for patients with less than 12 years of education, one point was added to the total score for each year of education not completed) (Grewal et al., 2016). The evaluation of specific cognitive subdomains was conducted using a standardized neuropsychological battery. Visuospatial ability was assessed with the Clock Drawing Test (CDT) (Ricci et al., 2016), executive function with the Trail Making Test A (TMT-A) (Bondi et al., 2014), learning and memory with the Hopkins Verbal Learning Test-Revised (HVLT-R) (Hammers et al., 2022), and language function with the Boston Naming Test (BNT) (Ellendt et al., 2017). Patients were stratified into a VCI group and a normal cognition (NC) group. The VCI group included patients who met the cognitive impairment threshold on either the MMSE or MoCA or who demonstrated impaired performance in any of the four tests (CDT, TMT-A, BNT or HVLT-R), even with normal global screening scores. The NC group comprised individuals with normal scores on both the MMSE and the MoCA, as well as all four subdomain tests.

### 2.4 Blood biomarker examination

All patients were required to have venous blood collected from 6:00 to 7:00 after 8 h of fasting. Blood samples were mixed with 2 mL of EDTA anticoagulant, and a Sysmex XN-10 hematology analyzer (Japan) was used to detect the following indicators: complete blood count [red blood cell (RBC) count, leukocyte count, and platelet count], erythrocyte characteristics (hemoglobin concentration and mean corpuscular hemoglobin), and leukocyte differential (neutrophil count and lymphocyte count). Additionally, 5-mL blood samples were collected for comprehensive biochemical profiling. The data were analyzed with a Hitachi 7,600 automatic biochemical analyzer (Japan). The following categories of parameters were assessed: (1) general biochemistry, including homocysteine (Hcy), retinol binding protein, and ischemia-modified albumin levels; (2) renal function, including creatinine (Cr), CysC, urea nitrogen (UN), and uric acid (UA) levels; (3) liver function including prealbumin, 5′-nucleotidase, *γ*-glutamyl transpeptidase, total bilirubin, cholinesterase, lactic dehydrogenase, direct bilirubin, and indirect bilirubin levels; (4) lipid metabolism, including lipoprotein *α*, triglyceride, high-density lipoprotein cholesterol, apolipoprotein A1, apolipoprotein B, and low-density lipoprotein cholesterol levels; and (5) coagulation function, including fibrinogen (FIB) and D-dimer levels. All tests were performed with standardized commercial kits by professional certifiers. The specific detection methods were the same as those published in our previous study (Tao et al., 2023).

### 2.5 MRI and image analysis

Neuroimaging was conducted via a 1.5T Siemens scanner (MAGNETOM Trio, A Tim System, Germany). The imaging protocol involved T2-weighted, T1-weighted, fluid-attenuated inversion recovery and susceptibility-weighted sequences. On the basis of the established morphological criteria, two experienced senior specialists identified cerebral hemorrhage lesions, cerebral infarction lesions, WMH and brain atrophy. To ensure the reliability and reproducibility of the assessments, the inter-rater agreement was evaluated using Cohen’s kappa. WMH was defined as hyperintense foci on fluid-attenuated inversion recovery sequences and was categorized as either periventricular WMH (PWMH) or deep WMH (DWMH) (Wardlaw et al., 2013). The severity of each category was independently assessed according to a standard Fazekas score (0–3) (Prins and Scheltens, 2015). Fazekas scores ≤ 1 were defined as mild PWMH (mPWMH) or mild DWMH (mDWMH), and Fazekas scores > 1 were defined as severe PWMH (sPWMH) or severe DWMH (sDWMH). The total WMH burden was stratified based on the summed Fazekas scores for PWMH and DWMH. A total score of <3 served as the threshold for mild total WMH (mTWMH), whereas a score of ≥3 defined severe total WMH (sTWMH) (Rao et al., 2024).

### 2.6 Statistical analysis

Statistical analyses were conducted with SPSS statistics software (version 23.0, Chicago, IL, USA). We first assessed the distribution of continuous variables for normality, including age, BMI, and all blood biomarkers. Data are presented as the mean ± standard deviation for normally distributed variables and as the median (interquartile range) for nonnormally distributed variables. For comparisons between the VCI and NC groups, variables were analyzed using independent samples t tests for parametric continuous data, the Mann–Whitney U test for nonparametric continuous data, and the chi-square test for categorical variables. Correlations were evaluated with Pearson’s or Spearman’s correlation coefficients, which were selected on the basis of the data distribution. The diagnostic efficacy of the combined variables for VCI was determined via binary logistic regression, with the NC group designated as the control group.

## 3 Results

### 3.1 Demographic characteristics

Our study included 334 patients with CSVD. In accordance with the diagnostic criteria, the patients were classified into the VCI group (*n* = 172) or the NC group (*n* = 162). Comparative analysis revealed that patients in the VCI group were significantly older than those in the NC group (*t* = 6.175, *p* < 0.001). Furthermore, there was a greater proportion of patients with a history of stroke (*χ*^2^ = 5.333, *p* = 0.021) and coronary heart disease (*χ*^2^ = 4.449, *p* = 0.035) in the VCI group. In contrast, the groups did not differ significantly regarding other baseline characteristics, such as gender, BMI, years of education, hypertension status, diabetes mellitus status, alcohol intake history and smoking history (Table 1).

**Table 1:** Comparison of basic demographic data between the two groups of CSVD patients.

Variables		VCI (*n* = 172)	NC (*n* = 162)	*t / χ*^2^ */ Z*	*p*
Age (years)^a^		69.41 ± 9.94	62.73 ± 9.82	6.175	**<0.001** ^***^
Body mass index (kg/m^2^)^a^		23.80 ± 3.24	24.01 ± 2.95	0.354	0.724
Gender (male)^b^		73 (42.4)	69 (42.6)	0.001	0.978
Years of education^b^					
Classification	0	2 (1.2)	3 (1.9)	11.365	**0.010** ^*^
	1 ~ 6	37 (21.5)	52 (32.1)		
	7 ~ 11	108 (62.8)	72 (44.4)		
	≥ 12	25 (14.5)	35 (21.6)		
History of stroke^b^		35 (20.3)	18 (11.1)	5.333	**0.021** ^*^
Hypertension^b^		130 (75.6)	114 (70.4)	1.151	0.283
Diabetes mellitus^b^		64 (37.2)	52 (32.1)	0.961	0.327
Coronary heart disease^b^		45 (26.2)	27 (16.7)	4.449	**0.035** ^*^
Smoking history^b^		61 (35.5)	64 (39.5)	0.582	0.446
Alcohol intake history^b^		47 (27.3)	45 (27.8)	0.009	0.926

^*^p < 0.05, ^**^p < 0.01, ^***^p < 0.001.

VCI, vascular cognitive impairment; NC, normal cognition.

a Shown as the mean ± standard deviation.

b Shown as *n* (%).

### 3.2 Comparison of blood biomarkers between the VCI and the NC groups

**Figure 2:**
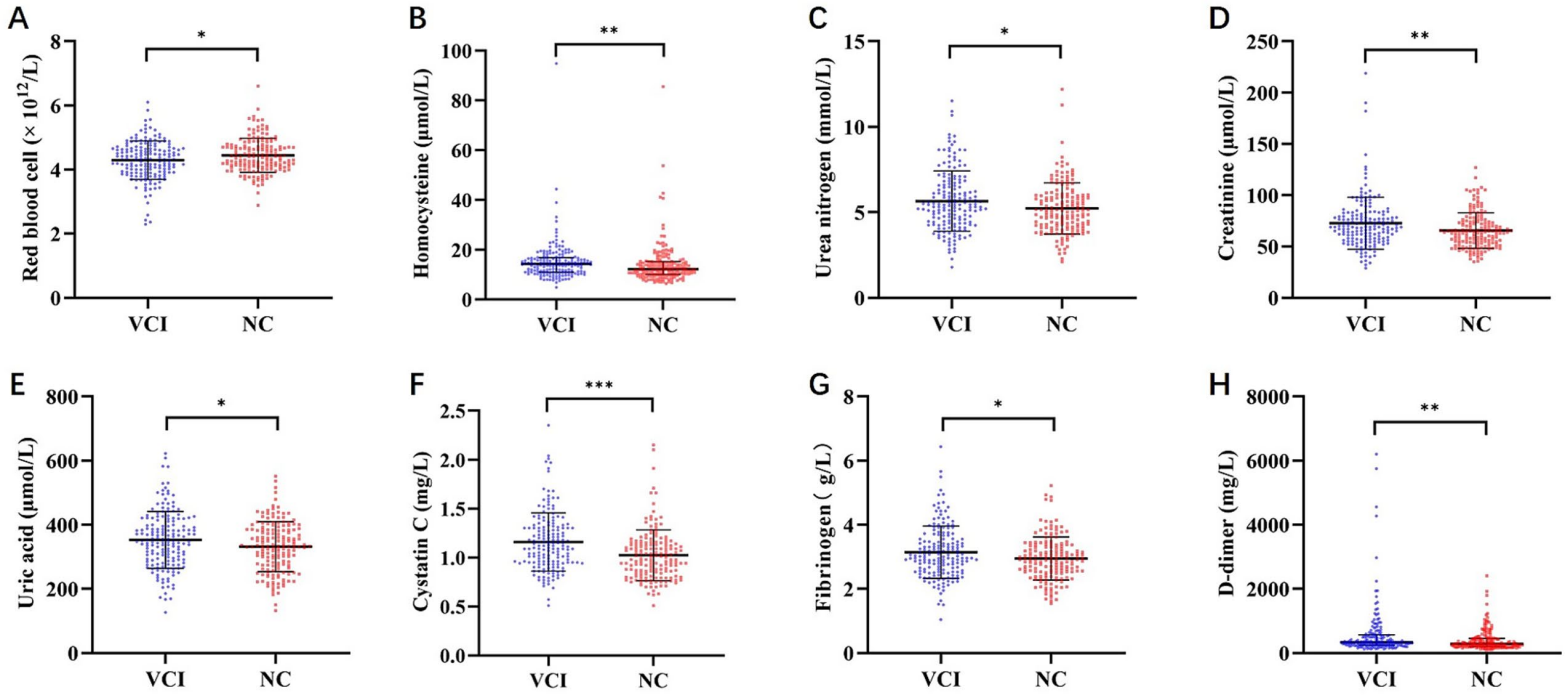
Comparison of blood biomarkers levels between the two groups of CSVD patients. The scatter plots show the serum concentrations of key biomarkers in patients with VCI compared to those with in the NC group. **(A–H)** present the following: **(A)** red blood cell count, **(B)** homocysteine levels, **(C)** urea nitrogen levels, **(D)** creatinine levels, **(E)** uric acid levels, **(F)** cystatin C levels, **(G)** fibrinogen levels, and **(H)** D-dimer levels. CSVD, cerebral small vessel disease; NC, normal cognition; VCI, vascular cognitive impairment.

Comparative analysis of hematological and biochemical profiles revealed several significant differences between the VCI and NC groups. The VCI group demonstrated a significant reduction in RBC count (*t* = 2.447, *p* = 0.015), eGFR (*t* = 3.579, *p* < 0.001) and a significant increase in Hcy levels (*t* = 2.993, *p* = 0.003). Multiple renal function parameters, including Cr (*t* = 2.976, *p* = 0.003), CysC (*t* = 4.428, *p* < 0.001), UN (*t* = 2.409, *p* = 0.017) and UA (*t* = 2.283, *p* = 0.023) levels, were significantly increased in the VCI group. The levels of indicators related to coagulation function, such as FIB (*t* = 2.445, *p* = 0.015) and D-dimer (*t* = 2.776, *p* = 0.006), were also significantly higher in the VCI group. No significant intergroup differences were found in the comprehensive panel of all other biomarkers (all *p* > 0.05). These included measurements of hemoglobin, complete blood count components (neutrophil, lymphocyte, and leukocyte), hepatic enzymes (5′-nucleotidase, cholinesterase, *γ*-glutamyl transpeptidase, and lactic dehydrogenase), lipid metabolism markers (lipoprotein *α*, triglycerides, high-density and low-density lipoprotein cholesterol, and apolipoprotein A1 and B), retinol binding protein, prealbumin, ischemia-modified albumin, total/direct/indirect bilirubin, and mean corpuscular hemoglobin (Table 2; Figure 2).

**Table 2:** Comparison of blood biomarkers levels between the two groups of CSVD patients.

Variables	VCI (*n* = 172)	NC (*n* = 162)	*t/Z*	*p*
Red blood cell (×10^12^/L)^a^	4.29 ± 0.60	4.44 ± 0.53	2.447	**0.015** ^*^
Hemoglobin (g/L)^a^	131.33 ± 17.79	132.73 ± 15.19	0.771	0.441
Platelet (×10^9^/L)^a^	213.83 ± 64.40	215.34 ± 57.39	0.226	0.821
Neutrophils (×10^9^/L)^a^	4.05 ± 1.44	3.78 ± 1.29	1.777	0.076
Lymphocyte (×10^9^/L)^a^	1.69 ± 0.56	1.78 ± 0.54	1.558	0.120
Leucocyte (×10^9^/L)^a^	6.42 ± 1.66	6.22 ± 1.56	1.099	0.273
Homocysteine (μmol/L)^b^	14.26 (5.82)	12.13 (5.11)	2.993	**0.003** ^**^
Retinol binding protein (mg/L)^a^	45.00 ± 12.43	45.41 ± 13.06	0.293	0.770
Urea nitrogen (mmol/L)^a^	5.65 ± 1.76	5.22 ± 1.50	2.409^*^	**0.017** ^*^
Creatinine (μmol/L)^a^	72.62 ± 25.21	65.60 ± 17.37	2.976^*^	**0.003** ^**^
eGFR^a^	92.60 ± 20.48	84.20 ± 22.28	3.579	**<0.001** ^***^
Uric acid (μmol/L)^a^	352.51 ± 88.56	331.65 ± 77.67	2.283	**0.023** ^*^
Cystatin C (mg/L)^a^	1.16 ± 0.30	1.02 ± 0.26	4.428^*^	**< 0.001** ^***^
5′-Nucleotidase (U/L)^a^	4.83 ± 2.24	4.89 ± 2.25	0.228	0.820
GGT(U/L)^b^	22.10 (15.63)	23.45 (17.00)	0.216	0.829
Cholinesterase (U/L)^a^	8122.37 ± 899.83	8430.57 ± 702.07	1.558	0.120
Lactic dehydrogenase (U/L)^a^	180.42 ± 35.64	173.92 ± 31.44	1.770	0.079
Triglycerides (mmol/L)^b^	1.33 (0.99)	1.41 (1.17)	0.306	0.760
HDL-C (mmol/L)^a^	1.15 ± 0.30	1.13 ± 0.28	0.632	0.528
LDL-C (mmol/L)^a^	2.59 ± 0.83	2.64 ± 0.89	0.526	0.599
Lipoprotein a (mg/L)^b^	154.46 (217.11)	131.16 (172.37)	1.192	0.233
Apolipoprotein A1 (g/L)^a^	1.14 ± 0.22	1.13 ± 0.22	0.492	0.623
Apolipoprotein B (g/L)^a^	0.79 ± 0.21	0.81 ± 0.21	0.726	0.468
IMA (U/mL)^a^	73.74 ± 5.99	73.05 ± 6.46	1.010	0.313
Prealbumin (mg/L)^a^	238.30 ± 53.63	248.08 ± 54.72	1.650	0.100
Total bilirubin (μmol/L)^a^	12.22 ± 5.26	11.71 ± 4.66	0.944	0.346
Direct bilirubin (μmol/L)^a^	4.11 ± 1.95	3.79 ± 1.71	1.616	0.107
Indirect bilirubin (μmol/L)^a^	8.12 ± 3.61	7.97 ± 3.31	0.411	0.682
Fibrinogen (g/L)^a^	3.14 ± 0.81	2.94 ± 0.67	2.445^*^	**0.015** ^*^
D-dimer (mg/L)^b^	330.00 (326.5)	280.00 (250.50)	2.776	**0.006** ^**^
Mean corpuscular hemoglobin^b^	30.50 (2.16)	30.40 (2.03)	1.649	0.099

^*^p < 0.05, ^**^p < 0.01, ^***^p < 0.001.

VCI, vascular cognitive impairment; NC, normal cognition; GGT, γ-glutamyl transpeptidase; HDL-C, high-density lipoprotein cholesterol; LDL-C, low-density lipoprotein cholesterol.

a Shown as the mean ± standard deviation.

b Shown as the median (IQR).

### 3.3 Comparison of cognitive behavioral scores between the VCI and the NC groups

**Figure 3:**
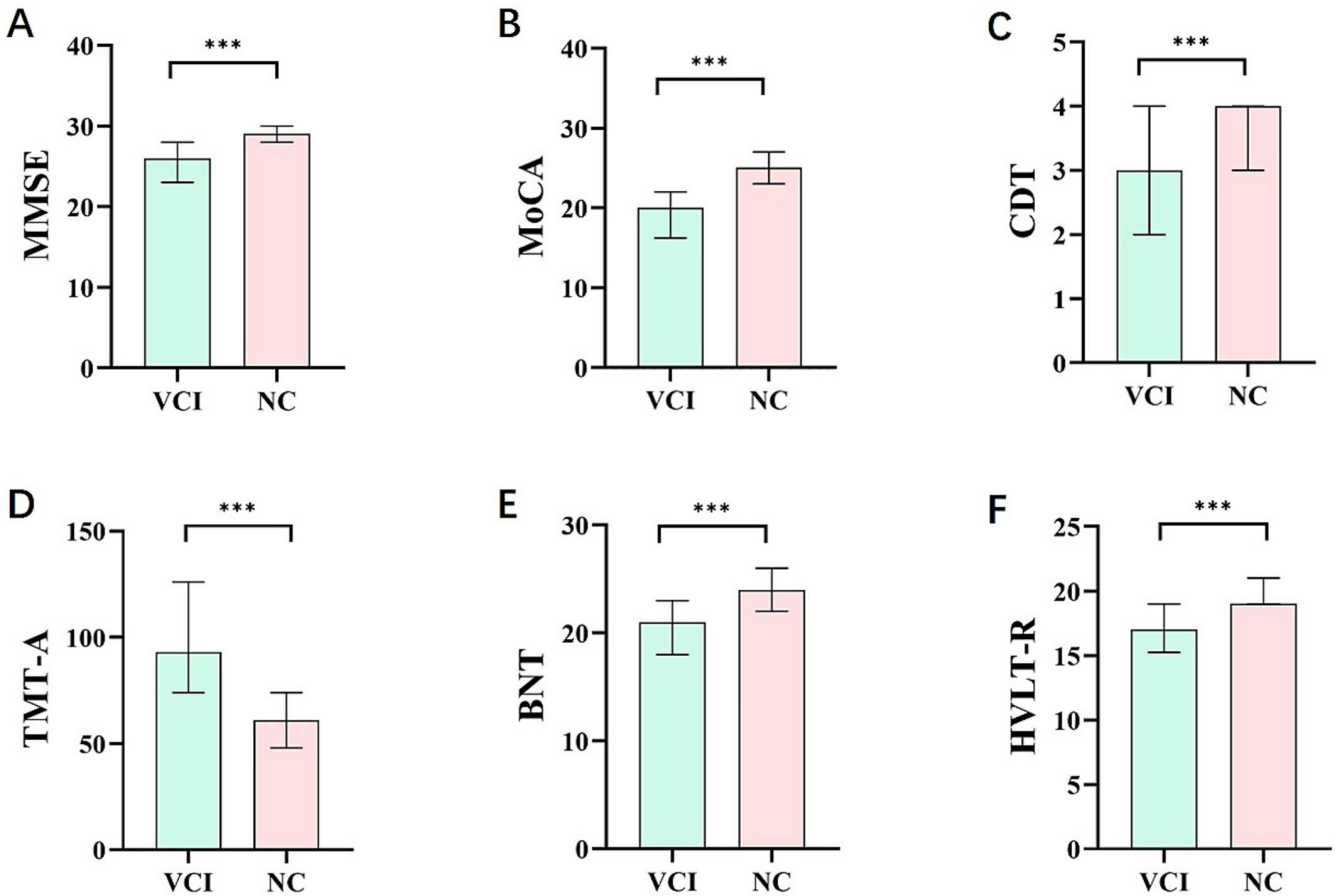
Comparison of cognitive behavioral scores between two groups of CSVD patients. The column bar graphs show cognitive test scores for patients with VCI and those in the NC group. **(A,B)** present differences global cognitive function scores between the two groups (MMSE and MoCA). **(C–F)** present differences in domain-specific neuropsychological test scores between the two groups (CDT, TMT-A, BNT, and HVLT-R). CSVD, cerebral small vessel disease; NC, normal cognition; VCI, vascular cognitive impairment; MMSE, Mini-Mental State Examination; MoCA, Montreal Cognitive Assessment; CDT, Clock Drawing Test; TMT-A, Trail-Making Test-A; BNT, Boston Naming Test; HVLT-R, Hopkins Verbal Learning Test-Revised.

As anticipated, compared with the NC group, the VCI group demonstrated significantly poorer performance across all the cognitive measures. Specifically, the VCI group exhibited notable deficits in global cognitive screens, with lower scores on both the MoCA (*z* = 12.664, *p* < 0.001) and the MMSE (*z* = 9.758, *p* < 0.001). Significant impairments were also observed in domain-specific tests, including the CDT (*z* = 7.024, *p* < 0.001), TMT-A (*z* = 10.720, *p* < 0.001), BNT (*z* = 8.300, *p* < 0.001) and HVLT-R (*z* = 7.771, *p* < 0.001) (Table 3; Figure 3).

**Table 3:** Comparison of cognitive behavioral scores between the two groups of CSVD patients.

Variables	VCI (*n* = 172)	NC (*n* = 162)	*Z*	*p*
Mini Mental State Examination	26.00 (5.00)	29.00 (2.00)	9.758	**<0.001** ^***^
Montreal cognitive assessment	20.00 (6.00)	25.00 (4.00)	12.664	**<0.001** ^***^
Clock Drawing Test	3.00 (2.00)	4.00 (1.00)	7.024	**<0.001** ^***^
Trail Making Test-A	93.00 (52.00)	61.00 (26.00)	10.720	**<0.001** ^***^
Boston Naming Test	21.00 (5.00)	24.00 (4.00)	8.300	**<0.001** ^***^
Hopkins Verbal Language Learning Test-Revised	17.00 (4.00)	19.00 (2.00)	7.771	**<0.001** ^***^

^*^p < 0.05, ^**^p < 0.01, ^***^p < 0.001. VCI, vascular cognitive impairment; NC, normal cognition.

### 3.4 Comparison of imaging features between the VCI and NC groups

**Figure 4:**
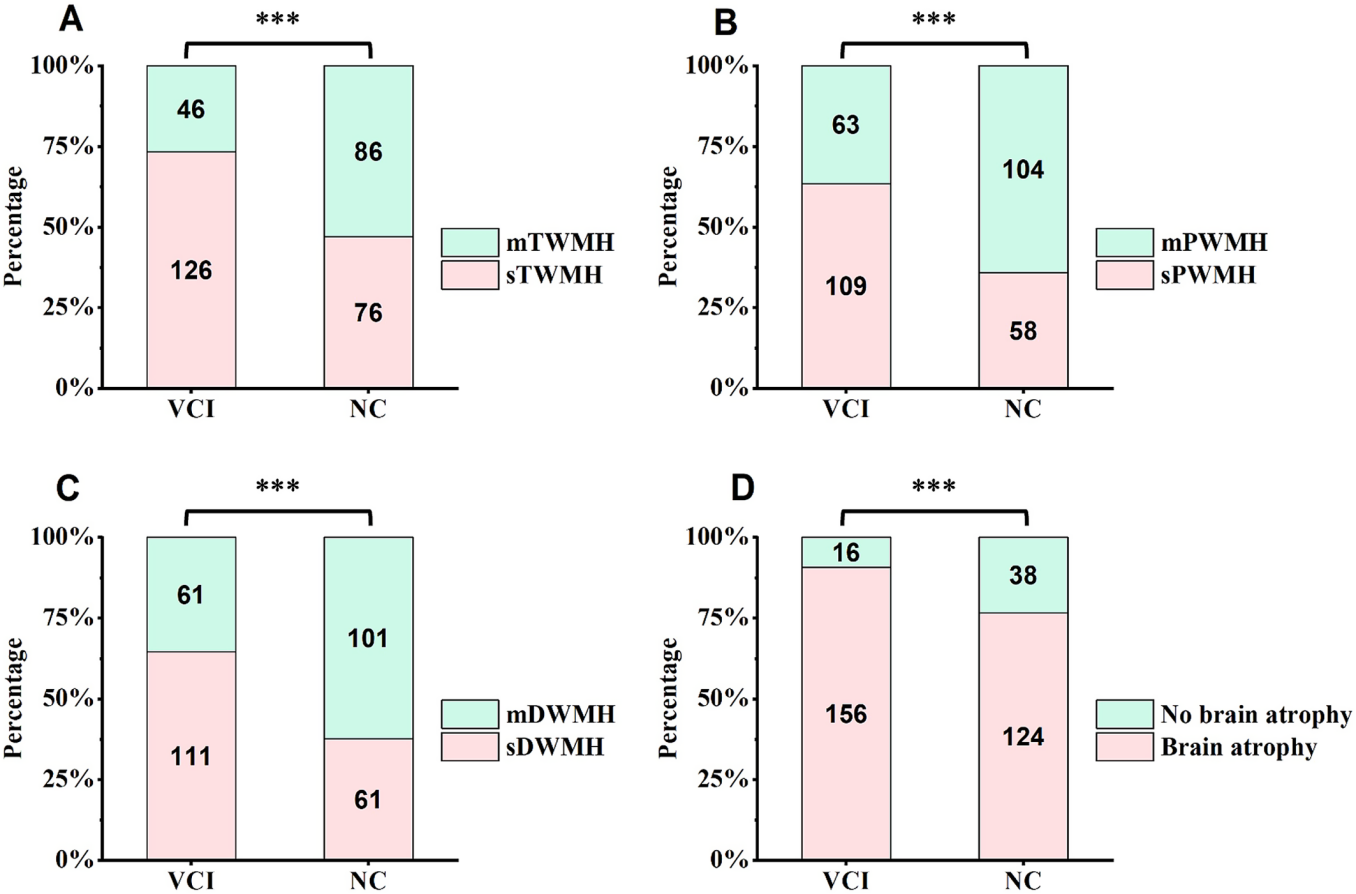
Comparison of imaging features between two groups of CSVD patients. The stacked bar charts show the percentage of key imaging markers in patients with VCI and those with NC. **(A–C)** present differences in the proportions of sTWMH, sPWMH, and sDWMH between the two groups, **(D)** shows the difference in the incidence of brain atrophy between the two groups. The numbers within each bar represent the actual number of patients classified into each imaging category. CSVD, cerebral small vessel disease; VCI, vascular cognitive impairment; NC, normal cognition; sTWMH, severe total white matter hyperintensity; sPWMH, severe periventricular white matter hyperintensity; sDWMH, severe deep white matter hyperintensity.

Compared with that in the NC group, the WMH burden in the VCI group was higher, with significant increases in the proportions of sTWMH (*χ*^2^ = 23.841, *p* < 0.001), sPWMH (*χ*^2^ = 25.364, *p* < 0.001) and sDWMH (*χ*^2^ = 24.134, *p* < 0.001). The incidence of brain atrophy was also significantly greater in the VCI group (*χ*^2^ = 12.332, *p* < 0.001). In contrast, the incidence of cerebral hemorrhage, cerebral infarction and infratentorial stroke did not differ significantly between the two groups (Table 4; Figure 4).

**Table 4:** Comparison of imaging features between the two groups of CSVD patients.

Variables	VCI (*n* = 172)	NC (*n* = 16)	*χ*^2^ *p*	*p*
sTWMH *n* (%)	126 (73.3)	76 (46.9)	24.220	**<0.001** ^***^
sPWMH *n* (%)	109 (63.4)	58 (35.8)	25.364	**<0.001** ^***^
sDWMH *n* (%)	111 (64.5)	61 (37.7)	24.134	**<0.001** ^***^
Brain atrophy *n* (%)	156 (90.7)	124 (76.5)	12.332	**<0.001** ^***^
Cerebral hemorrhage *n* (%)	3 (1.7)	3 (1.9)	0.005	0.941
Cerebral infarction *n* (%)	49 (28.5)	41 (25.3)	0.428	0.513
Infratentorial stroke *n* (%)	20 (11.6)	15 (9.3)	0.499	0.480

^*^p < 0.05, ^**^p < 0.01, ^***^p < 0.001. VCI, vascular cognitive impairment; NC, normal cognition; sDWMH, severe deep white matter hyperintensity; sPWMH, severe periventricular white matter hyperintensity; sTWMH, severe white matter hyperintensity.

### 3.5 Correlations between blood-based biomarkers and cognitive performance in all CSVD patients

Multiple blood indicators were significantly associated with cognitive or subdomain scores in our CSVD cohort. A positive correlation was observed between RBC count and global cognition, as measured by both the MMSE (*r* = 0.223, *p* < 0.001) and the MoCA (*r* = 0.244, *p* < 0.001). Specific subdomain analyses also revealed significant associations between RBC count and CDT (*r* = 0.161, *p* = 0.003) and BNT (*r* = 0.129, *p* = 0.018) scores. Conversely, a higher RBC count was associated with better processing speed and executive function, as showed by a negative correlation with TMT-A completion time (*r* = −0.249, *p* < 0.001).

We subsequently investigated the relationships between additional serum biomarkers and cognitive scores. The analysis revealed a consistent pattern of inverse associations. Elevated Hcy levels were significantly negatively correlated with both the MMSE (*r*_s_ = −0.200, *p* < 0.001) and the MoCA (*r*_s_ = −0.166, *p* = 0.002) scores. Among the renal markers, various negative associations were observed: UN, Cr, and CysC levels were correlated with poorer global cognition, with CysC demonstrating the most robust effects (all *p* < 0.01). Furthermore, UN and CysC levels were significantly associated with poorer performance for the CDT and BNT. This pattern extended to coagulation parameters, where both FIB and D-dimer levels were significantly associated with worse global and domain-specific (CDT and BNT) cognitive performance. Slower performance in the TMT-A, which reflects reduced processing speed, was consistently linked to elevated levels of specific biomarkers, including Hcy, UN, Cr, CysC, FIB, and D-dimer (all *p* < 0.01) (Table 5).

**Table 5:** Correlation analysis between serum biomarkers levels and cognitive behavioral scores in all CSVD patients.

Variables		MMSE	MoCA	CDT	TMT-A	BNT	HVLT-R
RBC	*r*	0.223	0.244	0.161	−0.249	0.129	0.088
	*p*	**<0.001** ^***^	**<0.001** ^***^	**0.003** ^**^	**<0.001** ^***^	**0.018** ^*^	0.109
Hcy	*r*_s_	−0.200	−0.166	0.044	0.199	−0.088	−0.028
	*p*	**<0.001** ^***^	**0.002** ^**^	0.422	**<0.001** ^***^	0.108	0.611
UN	*r*	−0.221	−0.208	−0.111	0.264	−0.157	−0.107
	*p*	**<0.001** ^***^	**<0.001** ^***^	**0.043** ^*^	**<0.001** ^***^	**0.004** ^**^	0.052
Cr	*r*	−0.225	−0.164	0.009	0.163	−0.058	0.001
	*p*	**<0.001** ^***^	**0.003** ^**^	0.866	**<0.001** ^***^	0.289	0.984
UA	*r*	−0.090	−0.085	−0.029	0.073	0.012	0.049
	*p*	0.099	0.120	0.603	0.185	0.831	0.369
CysC	*r*	−0.284	−0.285	−0.098	0.272	−0.117	−0.076
	*p*	**<0.001** ^***^	**<0.001** ^***^	0.073	**<0.001** ^***^	**0.033** ^*^	0.168
FIB	*r*	−0.204	−0.202	−0.207	0.186	−0.109	0.033
	*p*	**<0.001** ^***^	**<0.001** ^***^	**<0.001** ^***^	**0.001** ^**^	**0.047** ^*^	0.547
D-Dimer	*r*_s_	−0.209	−0.211	−0.213	0.260	−0.093	−0.061
	*p*	**<0.001** ^***^	**<0.001** ^***^	**<0.001** ^***^	**<0.001** ^***^	0.090	0.269

^*^p < 0.05, ^**^p < 0.01, ^***^p < 0.001. RBC, red blood cell; Hcy, homocysteine; UN, urea nitrogen; Cr, creatinine; UA, uric acid; CysC, cystatin C; FIB, fibrinogen. MMSE, Mini-Mental State Examination; MoCA, Montreal Cognitive Assessment; CDT, Clock Drawing Test; TMT-A, Trail Making Test-A; BNT, Boston Naming Test; HVLT-R, Hopkins Verbal Language Learning Test-Revised.

### 3.6 Diagnostic VCI models and multivariate logistic regression analysis

The differential variables (RBC, Hcy, UN, Cr, UA, CysC, FIB, and D-dimer) of the two groups were used as references to construct a diagnostic model of VCI (Model 1). The model demonstrated good fit (*χ*^2^ = 13.068, *p* = 0.110) and achieved discriminatory performance with 67.9% sensitivity, 57.6% specificity and 62.6% overall diagnostic accuracy. On the basis of Model 1, another 8 differential variables (sDWMH, sPWMH, sWMH, cerebral atrophy, age, years of education, history of stroke, and history of coronary heart disease) were added to construct Model 2 (first adjusted). The results showed that the model also fit well (*χ*^2^ = 9.491, *p* = 0.303). Its discriminative performance yielded a better sensitivity of 69.8% and a greater specificity of 68.6%, and the diagnostic accuracy of the model was 69.2%. In this cohort of CSVD patients, cognitive dysfunction was independently linked to increased age (Odds Ratio [OR] = 1.067, *p* < 0.001), the presence of sTWMH (OR = 2.713, *p* < 0.001) and elevated cystatin C levels (OR = 2.677, *p* = 0.041). Finally, on the basis of Model 2, we adjusted for common possible factors that may affect cognition. After adjustment for gender, BMI, smoking history, alcohol intake history, hypertension status and diabetes mellitus status, the fit of Model 3 was still good (*χ*^2^ = 4.489, *p* = 0.810). Its predictive performance remained substantial, with a sensitivity of 71.0% and a specificity of 72.1%. Parameter estimation confirmed that age (OR = 1.064, *p* < 0.001) and sTWMH (OR = 2.695, *p* < 0.001) were significantly correlated with cognitive impairment. Importantly, after comprehensive adjustment, the CysC level (OR = 3.257, *p* = 0.021) emerged as the only blood biomarker that maintained an independent association with cognitive impairment (Table 6). To further validate the robustness of our findings, we conducted sensitivity analyses using eGFR in place of Cr. To avoid collinearity, age was excluded from the adjustment set for Model 2, and gender was excluded from the adjustment set for Model 3, while all other covariates remained consistent with the primary models. Across all these analyses, CysC consistently remained a robust blood biomarker independently associated with cognitive impairment (Model 1: OR = 4.689, *p* = 0.001; Model 2: OR = 2.677, *p* = 0.041; Model 3: OR = 3.257, *p* = 0.021) (Supplementary Table S1).

**Table 6:** Multivariate logistic regression analysis of the diagnostic value of blood markers in CSVD-CI.

Variables	Model 1		Model 2		Model 3	
	OR (95% CI)	*p*	^#^OR (95% CI)	*p*	^##^OR (95% CI)	*p*
CysC	4.689 (1.922, 11.438)	**0.001** ^**^	2.677 (1.042, 6.879)	**0.041** ^*^	3.257 (1.192, 8.899)	**0.021** ^*^
RBC	0.701 (0.463, 1.059)	0.092	0.845 (0.513, 1.391)	0.507	0.999 (0.578, 1.725)	0.996
Hcy	0.991 (0.962, 1.022)	0.572	0.983 (0.951, 1.015)	0.285	0.986 (0.954, 1.020)	0.414
UN	1.038 (0.886, 1.218)	0.642	0.994 (0.836, 1.183)	0.950	0.985 (0.822, 1.181)	0.872
Cr	1.001 (0.984, 1.018)	0.939	1.001 (0.998, 1.004)	0.467	1.011 (0.991, 1.032)	0.293
UA	1.002 (0.999, 1.005)	0.228	1.001 (0.987, 1.022)	0.651	1.001 (0.998, 1.005)	0.480
FIB	1.314 (0.959, 1.801)	0.089	1.293 (0.910, 1.838)	0.152	1.302 (0.903, 1.876)	0.157
D-dimer	1.000 (1.000, 1.001)	0.101	0.845 (0.513, 1.391)	0.507	0.999 (0.578, 1.725)	0.996

^*^p < 0.05, ^**^p < 0.01, ^***^p < 0.001. RBC, red blood cell; Hcy, homocysteine; UN, urea nitrogen; Cr, creatinine; UA, uric acid; FIB, fibrinogen. ^#^indicates that the differential variables in the univariate analysis, such as sDWMH, sPWMH, sTWMH, brain atrophy, age, years of education, history of stroke and coronary heart disease were adjusted. ^##^indicates that gender, BMI, smoking history, alcohol intake history, hypertension and diabetes mellitus were adjusted based on Model 2.

### 3.7 Receiver operating characteristic (ROC) curves of the VCI models

**Figure 5:**
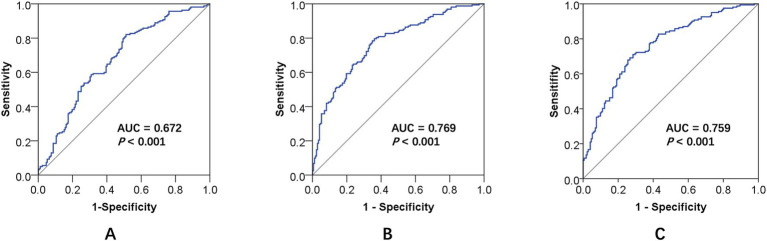
ROC curve comparison of three predictive models for identifying cognitive impairment in patients with CSVD. **(A–C)** represent Model 1, Model 2, and Model 3, respectively. ROC, receiver operating characteristic; CSVD, cerebral small vessel disease; AUC, area under the curve.

The discriminatory performance of each model was evaluated using ROC curve analysis. Owing to its combined variables, Model 1 yielded an area under the curve (AUC) of 0.672 (95% Confidence Interval [CI]: 0.615 ~ 0.730, *p* < 0.001). The optimal cutoff was 0.550, corresponding to a sensitivity of 67.9%, a specificity of 57.6%, and a Youden index of 0.309 (Figure 5A). Model 2 demonstrated superior predictive power, with a significantly greater AUC of 0.769 (95% CI: 0.718 ~ 0.819, *p* < 0.001). At its optimal cutoff of 0.580, this model achieved a sensitivity of 79.6% and a specificity of 64.0%, resulting in a Youden index of 0.436 (Figure 5B). Similarly, Model 3 demonstrated high discriminatory capacity, with an AUC of 0.759 (95% CI: 0.708 ~ 0.810, *p* < 0.001). The optimal cutoff for this model was 0.502, with a sensitivity of 71.0% and a higher specificity of 71.5%, yielding a Youden index of 0.425 (Figure 5C). These models with multiple combined variables can effectively increase the efficacy of diagnosing cognitive dysfunction in patients with CSVD.

## 4 Discussion

This investigation reveals the following findings: (1) RBC, Hcy, FIB, D-dimer, UN, Cr, UA and CysC levels are associated with cognitive impairment in patients with CSVD, and (2) the serum CysC is an independent associated factor, underscoring its particular significance within the context of CSVD-related cognitive decline.

RBCs play key roles in maintaining brain tissue oxygenation and microcirculatory homeostasis. Previous studies have established that chronic hypoperfusion and microcirculatory dysfunction are critical mechanisms in the pathogenesis of CSVD (Wardlaw et al., 2019). Abnormal RBC counts or functions may exacerbate cerebral small vessel hemodynamic disturbances, resulting in white matter damage and ischemic neuronal injury. Our earlier study revealed that the RBC count is an independent correlate of VCI in patients with severe WMH (Tao et al., 2022). Subsequent studies further revealed that RBC count is an independent factor protecting cognitive function following nondisabling ischemic cerebrovascular events (Li et al., 2022). This study revealed both a significant decrease in the RBC count in CSVD-CI patients and a positive correlation between RBC count and cognitive scores across the CSVD cohort. Collectively, these findings indicate that RBC count may reflect cognitive impairment in patients with CSVD, thereby corroborating and extending the findings of previous reports (Li et al., 2022; Tao et al., 2022).

Hcy contributes to CSVD progression through inducing vascular endothelial dysfunction, disrupting the blood–brain barrier, and promoting neuroinflammatory responses and oxidative stress (Li et al., 2021). Numerous clinical studies have confirmed that elevated serum Hcy levels are a significant risk factor for both CSVD and cognitive impairment (Liao et al., 2025). Furthermore, Hcy levels are directly correlated with the radiological burden of CSVD and the degree of cognitive decline (Ji et al., 2020). Building on these associations, a mediation analysis revealed that the adverse effect of hyperhomocysteinemia on cognition was partially mediated through its contribution to specific CSVD imaging markers, such as WMH and lacunar infarcts (Teng et al., 2022). Moreover, an elevated Hcy level has been recognized as an independent predictor of cognitive deterioration or dementia conversion in CSVD patients (Cao and Sun, 2022). In this study, the Hcy concentration was significantly greater in the VCI group than in the NC group and was negatively correlated with the cognitive scores. Collectively, the results indicate that an elevated Hcy level is a significant and actionable contributor to cognitive decline in patients with CSVD, which has important implications for clinical management.

FIB, as a key mediator of the coagulation process, activates the coagulation cascade following endothelial injury (Foley and Conway, 2016; Levi et al., 2004). FIB directly contributes to microthrombus formation, obstructing cerebral small vessels and leading to white matter ischemia. Additionally, FIB induces monocyte infiltration and the release of inflammatory factors via β_2_ integrin receptor-dependent pathways, exacerbating vascular wall damage (Flick et al., 2004). The level of D-dimer, a marker of fibrin degradation, is elevated in response to microthrombus formation or secondary fibrinolysis. Through NADPH oxidase activation, D-dimers induce oxidative stress and neuroinflammation, leading to blood–brain barrier disruption, a greater incidence of white matter lesions and accelerated cognitive decline (Lominadze et al., 2005; Levi et al., 2004; Foley and Conway, 2016). Increased vascular shear stress due to hypertension, metabolic toxicity and neuroinflammation associated with diabetes collectively promotes FIB deposition and D-dimer release, ultimately resulting in the establishment of a vicious cycle of thrombosis, inflammation, and endothelial damage (Webb and Werring, 2022; Maida et al., 2022). The roles of FIB and D-dimer in CSVD, particularly their potential as biomarkers for tracking disease progression, have been extensively investigated. Recent clinical evidence of subcortical infarctions has indicated that elevated D-dimer levels are associated with a greater neuroimaging burden (Hao et al., 2024), whereas higher FIB levels are correlated with a higher prevalence of asymptomatic cerebrovascular lesions (Aono et al., 2007). Thus, both D-dimer and FIB levels act as independent factors for predicting greater WMH severity in patients with CSVD (Xu et al., 2024). In addition to structural damage, D-dimer levels serve as important predictors of cognitive decline among community-based elderly individuals, with increased levels corresponding to increased risk of cognitive decline (Aono et al., 2007). Furthermore, elevated concentrations of both FIB and D-dimer increase the risk of vascular dementia (Zhou et al., 2020; Quinn et al., 2011). Consistent with previous research, this study revealed that FIB and D-dimer levels were significantly higher in patients with cognitive impairment, and that these levels were negatively correlated with cognitive scores.

The kidney and brain, as high-perfusion terminal organs, share similar microvascular structures and blood flow regulatory mechanisms and are particularly susceptible to small vessel pathology in the context of common predisposing conditions such as hypertension and diabetes (Ito et al., 2009). A robust link between renal dysfunction and CSVD has been extensively documented. A large-scale meta-analysis established a notable link between albuminuria levels and the imaging hallmarks of CSVD (Georgakis et al., 2018). A reduced eGFR has been linked to more severe WMH, enlarged perivascular spaces, a higher incidence of lacunar lesions, brain atrophy and cerebral microbleeds, and a greater total imaging load (Nash and Fandler-Höfler, 2024; Xiao et al., 2015). Moreover, a decreased GFR is significantly correlated with cognitive decline (Kurella Tamura et al., 2008) and is mediated by vascular risk factors, inflammation, oxidative stress, Aβ deposition and brain atrophy (Mazumder et al., 2019). CysC is a sensitive marker for evaluating the eGFR and is unaffected by age and muscle mass (Coll et al., 2000); additionally, compared with the Cr-based eGFR, the CysC-based eGFR has greater value for predicting the risk of cognitive impairment (Paterson et al., 2021). Meta-analyses have reported that elevated serum CysC levels are significantly correlated with a higher incidence of mild cognitive impairment, particularly in Asian populations (Nair et al., 2020). Longitudinal cohort studies have confirmed the dose–effect relationship of elevated serum CysC levels and cognitive decline in elderly individuals (Sarnak, 2008; Cui et al., 2020; Yaffe et al., 2008). Although the UN and UA are not direct measures of the eGFR, elevated UA levels have been associated with CSVD and deficits in attention and executive function (Tang et al., 2022), whereas increased UN levels may increase the risk of cerebral microbleeds (Liu et al., 2024). Our findings revealed that the serum concentrations of UN, Cr, UA, and CysC significantly increased in the VCI group. These elevated biomarker levels showed significant inverse correlations with cognitive scores, indicating their association with CSVD-CI.

Notably, CysC itself has neuroprotective effects, and its pathological elevation leads to negative outcomes. This phenomenon should be interpreted from the perspective of dynamic pathological processes. In the early stages of CSVD, physiological elevation of CysC inhibits CatB, thereby reducing neuronal apoptosis (Kaur et al., 2010). In this process, CysC binds to soluble Aβ and delays amyloidogenesis (Tizon et al., 2010). As microvascular damage progresses, owing to the persistently elevated CysC levels, CysC competitively occupies the active site of CatB, impairing Aβ degradation and causing abnormal Aβ accumulation in the brain (Sun et al., 2008). Therefore, CysC elevation is also considered relevant to the onset and diagnosis of AD. As a fundamental driver of VCI, CSVD induces microvascular damage, which compromises blood–brain barrier integrity, facilitating the influx of circulating CysC into the central nervous system. Alternatively, this may trigger a compensatory increase in CysC levels due to chronic hypoxia. Both pathways ultimately converge to accelerate cognitive decline. This study confirms that CysC levels are independently associated with CSVD-CI, suggesting their potential as an early biomarker for screening this condition. Moreover, given the dual role of CysC in both vascular integrity and Aβ metabolism, the observed association may reflect overlapping vascular and neurodegenerative mechanisms. Unfortunately, this study lacks specific biomarkers (e.g., cerebrospinal fluid Aβ42/p-tau, amyloid PET scans, or even hippocampal volume measurements) to fully rule out early-stage AD.

Advancing age is a well-established risk factor for CSVD and its associated cognitive impairment (Inoue et al., 2023), and severe WMH represent a key imaging correlate of cognitive decline in CSVD (Prins and Scheltens, 2015). In this study, multivariate logistic regression analysis confirmed that age (OR = 1.064) and sTWMH (OR = 2.695) were associated with cognitive impairment in patients with CSVD. Notably, elevated serum CysC levels exhibited a robust and consistent association with cognitive impairment, as demonstrated by the unadjusted model (OR = 4.689) and sustained across two progressively adjusted models (OR = 2.677–3.257). Collectively, these findings suggest that CysC is independently associated with cognitive impairment in CSVD, supporting its potential value as a complementary indicator in clinical assessment.

## 5 Limitations

This study exhibits the following limitations. First, the sample size was limited, and the use of single-center data may introduce selection and information bias. Second, the observational nature of the study precluded causal inference and dynamic assessment, and confounding variables could not be fully excluded. Third, our analysis focused specifically on CysC levels in patients with age-related arteriolosclerotic CSVD, limiting the applicability of the findings to other subtypes. Future research should aim to: (1) establish risk stratification thresholds for CysC levels through a prospective cohort study considering race and baseline renal function; (2) investigate whether intensive control of hypertension and other vascular risk factors can improve the cognitive trajectory in patients with elevated CysC levels; (3) integrate advanced neuroimaging markers, including cerebral microbleeds, quantitative WMH burden; (4) include comparison groups with Alzheimer’s disease and mixed pathology; and (5) explore cerebrospinal fluid and blood biomarkers, such as glial fibrillary acidic protein, neurofilament light chain, Aβ and tau-related markers.

## 6 Conclusion

A composite panel of eight blood biomarkers significantly improves the diagnostic performance for VCI, with serum CysC independently associated with cognitive impairment in patients with CSVD.

## Data Availability

The raw data supporting the conclusions of this article will be made available by the authors, without undue reservation.

## Glossary

Aβ: amyloid β-protein
BMI: body mass index
BNT: Boston Naming Test
CatB: cathepsin B
CDT: Clock Drawing Test
Cr: creatinine
CSVD: cerebral small vessel disease
CSVD-CI: CSVD-related cognitive impairment
CysC: cystatin C
DWMH: deep white matter hyperintensity
FIB: fibrinogen
eGFR: estimated glomerular filtration rate
Hcy: homocysteine
HVLT-R: Hopkins Verbal Learning Test-Revised
mDWMH: mild deep white matter hyperintensity
MMSE: Mini-Mental State Examination
MoCA: Montreal Cognitive Assessment
mPWMH: mild periventricular white matter hyperintensity
MRI: magnetic resonance imaging
mTWMH: mild total white matter hyperintensity
NC: normal cognition
PWMH: periventricular white matter hyperintensity
RBC: red blood cell
sDWMH: severe deep white matter hyperintensity
sPWMH: severe periventricular white matter hyperintensity
sTWMH: severe total white matter hyperintensity
TMT-A: Trail-Making Test-A
UA: uric acid
UN: urea nitrogen
VCI: vascular cognitive impairment
WMH: white matter hyperintensity
