# Supplementary material for: Serum cystatin C levels are independently correlated with cognitive impairment in individuals with cerebral small vessel disease
Source: Front Neurosci. 2026 Apr 28;20:1781698. doi: 10.3389/fnins.2026.1781698 (PMC13160901; doi:10.3389/fnins.2026.1781698)
Supplement: Supplementary file 1 [file Supplementary_file_1.pdf]

Supplementary Material

Table S1 Multivariate logistic regression analysis of the diagnostic value of blood markers in CSVD-CI

Variables	Model 1		Model 2		Model 3	
	OR (95% CI)	*P*	#OR (95% CI)	*P*	##OR (95% CI)	*P*
CysC	4.689 (1.922, 11.438)	**0.001****	3.348 (1.311, 8.552)	**0.012***	4.319 (1.648, 11.320)	**0.003***
RBC	0.701 (0.463, 1.059)	0.092	0.612 (0.398, 0.942)	**0.026***	0.777 (0.471, 1.283)	0.324
Hcy	0.987 (0.957, 1.017)	0.391	0.993 (0.963, 1.025)	0.662	0.993 (0.961, 1.025)	0.643
UN	1.025 (0.876, 1.200)	0.760	1.023 (0.867, 1.207)	0.789	1.007 (0.850, 1.194)	0.934
eGFR	0.992 (0.977, 1.007)	0.296	1.001 (0.985, 1.018)	0.869	0.995 (0.978, 1.013)	0.590
UA	1.002 (0.999, 1.004)	0.319	1.001 (0.998, 1.005)	0.372	1.002 (0.998, 1.005)	0.365
FIB	1.314 (0.959, 1.801)	0.089	1.348 (0.969, 1.876)	0.076	1.336 (0.940, 1.900)	0.107
D-dimer	1.000 (1.000, 1.001)	0.101	1.000 (1.000, 1.001)	0.173	1.000 (1.000, 1.001)	0.187

$^{*}P < 0.05$, $^{**}P < 0.01$, $^{***}P < 0.001$. CysC, cystatin C; RBC, red blood cell; Hcy, homocysteine; UN, urea nitrogen; eGFR, estimated glomerular filtration rate; UA, uric acid; FIB, fibrinogen. # indicates that the differential variables in the univariate analysis, such as sDWMH, sPWMH, sTWMH, brain atrophy, age, years of education, history of stroke and coronary heart disease were adjusted. ## indicates that gender, BMI, smoking history, alcohol intake history, hypertension and diabetes mellitus were adjusted based on Model 2.
